# Incidence, reinfection, and reproductive morbidity of bacterial sexually transmitted infections among Kenyan adolescent girls and young women receiving systematic testing and treatment: a 4.5-Year prospective cohort analysis

**DOI:** 10.1016/j.lanafr.2026.100137

**Published:** 2026-11

**Authors:** Maricianah Onono, Kidist Zewdie, Elizabeth R. Brown, Nelly Mugo, George Otieno, Imeldah Wakhungu, Kate B. Heller, Elizabeth A. Bukusi, Ruanne V. Barnabas

**Affiliations:** a Center for Microbiology Research, Kenya Medical Research Institute (KEMRI), Kisumu, Kenya; b School of Public Health, University of Illinois Chicago, Chicago, IL, USA; c Center for Clinical Research, Kenya Medical Research Institute (KEMRI), Nairobi, Kenya; d Division of Infectious Diseases, Massachusetts General Hospital, Boston, MA, USA; e Department of Medicine, Harvard Medical School, Boston, MA, USA; f Department of Epidemiology, Harvard T.H. Chan School of Public Health, Boston, MA, USA

**Keywords:** Adolescent girls and young women (AGYW), Sexually transmitted infections (STIs), Asymptomatic infections, Reinfection, Syndromic management, Etiologic screening, Partner notification, Kenya

## Abstract

**Background:**

Sexually transmitted infections (STIs) remain a major burden among adolescent girls and young women (AGYW) in sub-Saharan Africa. We investigated STI incidence, reinfection, and reproductive morbidity in Kenyan AGYW receiving regular testing and treatment.

**Methods:**

We analysed data from the KEN SHE study, which enrolled sexually active, HIV-negative AGYW aged 15–20 years in Kenya. At enrolment and every six months, participants were tested for *Neisseria gonorrhoeae* (GC), *Chlamydia trachomatis (CT)* and syphilis. Testing used nucleic acid amplification for GC/CT and rapid plasma reagin with treponemal confirmation for syphilis. Symptomatic or laboratory-positive participants received treatment per national guidelines. Pelvic inflammatory disease (PID) was diagnosed and treated syndromically. Andersen-Gill recurrent events Cox models with time-varying covariates assessed factors associated with incidence and reinfection.

**Findings:**

Between December 2018 and November 2019, 2275 participants were enrolled and followed for a median of 54 months (interquartile range [IQR] 52–57). Overall, 1355 (60%) tested positive for at least one of GC, CT, or syphilis (72.5% asymptomatic). Overall incidence of GC/CT was 22.37 per 100 person-years (95% CI: 21.42–23.34), CT (19.06 per 100 person-years; 95% CI: 18.19–19.96), and GC (5.35 per 100 person-years; 95% CI: 4.89–5.83). Among those with GC/CT, 43% experienced at least one reinfection (25.20 per 100 person-years; 95% CI: 23.21–27.30). Overall, 13.4% (304/2275) received PID treatment; incident PID was 3.34 per 100 person-years (95% CI 2.97–3.75). Participants with untreated STIs had a PID incidence of 12.83 per 100 person-years (95% CI 9.90–16.30).

**Interpretation:**

GC and CT incidence, reinfection, and PID remained high despite systematic testing and treatment, indicating substantial residual disease burden. Partner management, vaccination and prophylaxis require prioritization and integration.

**Funding:**

Bill & Melinda Gates Foundation; Francis and Dorothea Reed Endowed Chair in Infectious Diseases.

## Muhtasari

**Utangulizi:**

Maambukizi ya zinaa (STIs) yanaendelea kuwa tatizo kubwa la afya miongoni mwa wasichana balehe na wanawake vijana (AGYW) katika Afrika Kusini mwa Jangwa la Sahara. Tulichunguza kiwango cha maambukizi mapya ya STIs, maambukizi ya kurudia, na matatizo ya afya ya uzazi miongoni mwa AGYW nchini Kenya waliokuwa wakipimwa na kutibiwa mara kwa mara.

**Mbinu:**

Tulichambua takwimu kutoka utafiti wa KEN SHE, uliowahusisha AGYW wanaofanya ngono, wasio na maambukizi ya virusi vya ukimwi (VVU), wenye umri wa miaka 15 hadi 20 nchini Kenya. Washiriki walipimwa maambukizi ya *Neisseria gonorrhoeae* (GC), bakteria anayesababisha kisonono, *Chlamydia trachomatis* (CT), bakteria anayesababisha klamidia, na kaswende. Vipimo vilifanyika kwa kutumia uchunguzi wa ukuzaji wa asidi ya nukleiki (nucleic acid amplification) kwa GC na CT, na kipimo cha Rapid Plasma Reagin (RPR) kilichothibitishwa kwa kipimo cha treponema kwa kaswende. Washiriki waliokuwa na dalili au waliopatikana na maambukizi kupitia vipimo vya maabara walitibiwa kulingana na miongozo ya kitaifa. Ugonjwa wa uvimbe wa nyonga (PID) ulitambuliwa na kutibiwa kwa kutumia mbinu ya utambuzi kulingana na dalili. Miundo ya Andersen-Gill ya Cox kwa matukio yanayojirudia, yenye viashiria vinavyobadilika kulingana na muda, ilitumika kutathmini sababu zilizohusishwa na maambukizi mapya na maambukizi ya kurudia.

**Matokeo:**

Kati ya Desemba 2018 na Novemba 2019, washiriki 2275 walijiunga na utafiti na walifuatiliwa kwa muda wa wastani wa miezi 54 (uwiano wa robo kati ya 52 hadi 57). Kwa ujumla, washiriki 1355 (asilimia 60) walipatikana na angalau moja kati ya GC, CT, au kaswende, na asilimia 72.5 hawakuwa na dalili. Kiwango cha maambukizi mapya ya GC au CT kilikuwa 22.37 kwa kila miaka 100 ya mtu (95% CI: 21.42 hadi 23.34), CT ilikuwa 19.06 kwa kila miaka 100 ya mtu (95% CI: 18.19 hadi 19.96), na GC ilikuwa 5.35 kwa kila miaka 100 ya mtu (95% CI: 4.89 hadi 5.83). Miongoni mwa waliopata GC au CT, asilimia 43 walipata angalau maambukizi moja ya kurudia (25.20 kwa kila miaka 100 ya mtu; 95% CI: 23.21 hadi 27.30). Kwa ujumla, asilimia 13.4 (304 kati ya 2275) walipokea matibabu ya PID. Kiwango cha PID mpya kilikuwa 3.34 kwa kila miaka 100 ya mtu (95% CI: 2.97 hadi 3.75). Washiriki waliokuwa na maambukizi ya zinaa ambayo hayakuwa yametibiwa walikuwa na kiwango cha PID cha 12.83 kwa kila miaka 100 ya mtu (95% CI: 9.90 hadi 16.30).

**Tafsiri:**

Viwango vya maambukizi mapya ya GC na CT, maambukizi ya kurudia, na ugonjwa wa uvimbe wa nyonga (PID) viliendelea kuwa vya juu licha ya upimaji na matibabu ya mara kwa mara. Hii inaonyesha kuwa mzigo wa magonjwa haya bado ni mkubwa. Huduma za kuwashirikisha na kuwatibu wenza wa ngono, matumizi ya chanjo, na hatua za kuzuia maambukizi zinapaswa kupewa kipaumbele na kuunganishwa katika huduma za afya.

**Ufadhili:**

Bill & Melinda Gates Foundation na Francis and Dorothea Reed Endowed Chair in Infectious Diseases.

**Research in context**

**Evidence before this study**

Sexually transmitted infections (STIs) remain a major public health burden among adolescent girls and young women (AGYW) in sub-Saharan Africa. We searched PubMed and Google Scholar for studies published between 1 January 2010 and 31 December 2024 using the terms “sexually transmitted infections”, “STI incidence”, “adolescent girls and young women”, “sub-Saharan Africa” and “Kenya”, with no language restrictions. Available data on STI burden among AGYW are largely derived from cross-sectional surveys, self-reports, single time-point testing or syndromic management, with limited longitudinal data describing incidence, reinfection rates and clinical outcomes. Few studies in sub-Saharan Africa have longitudinally evaluated whether intensive testing-and-treatment strategies, alongside syndromic management, can effectively control STI transmission and prevent reproductive sequelae in high-burden settings.

**Added value of this study**

This study provides one of the largest and longest longitudinal assessments of chlamydia, gonorrhoea, and syphilis incidence, reinfection, and reproductive sequelae among AGYW in sub-Saharan Africa. We followed 2275 sexually active young Kenyan women for a median of 54 months with systematic six-monthly laboratory testing and immediate treatment per Kenya national guidelines. We found that chlamydia and gonorrhoea persisted at high rates despite quarterly syndromic screening, six-monthly laboratory testing, immediate treatment, risk-reduction counselling and comprehensive sexual health services: 60% of participants tested positive for at least one of chlamydia, gonorrhoea, or syphilis; among the 1348 participants who had chlamydia or gonorrhoea, 43.2% experienced a first reinfection with the same pathogen; and 13.4% received treatment for pelvic inflammatory disease. Pelvic inflammatory disease incidence was particularly elevated among participants with laboratory-confirmed chlamydia or gonorrhoea who did not receive adequate treatment. As an observational study without a comparator arm, our findings describe the persistent burden under intensive case management rather than directly evaluating the testing-and-treatment paradigm.

**Implications of all the available evidence**

Our findings, together with existing evidence, suggest that testing and treatment alone are unlikely to be sufficient for STI control or for preventing reproductive morbidity in high-burden settings. Complementary prevention approaches such as strengthened partner management and consistent condom promotion should be prioritized alongside continued investment in case management. Biomedical prevention strategies including post-exposure prophylaxis, pre-exposure prophylaxis, and vaccination warrant continued investigation, but until evidence for these tools strengthens in cisgender women, the most actionable path remains the comprehensive case management package above. Future research and policy should shift focus from measuring STI incidence alone to evaluating impact on clinically meaningful outcomes, such as pelvic inflammatory disease, infertility, and other reproductive sequelae that disproportionately affect young women.

## Introduction

Globally, more than 1 million curable sexually transmitted infections (STIs) are acquired each day, totalling an estimated 374 million new cases of four curable STIs: *Treponema pallidum*, *Chlamydia trachomatis, Neisseria gonorrhoeae* and *Trichomonas vaginalis* in 2020.^1^^,^^2^ The global burden of STIs falls disproportionately on low-and middle-income regions, with Sub-Saharan Africa having the highest incidence rates.^3^ Adolescent girls and young women (AGYW) in sub-Saharan Africa are disproportionately affected and often harbour a high prevalence of asymptomatic bacterial STIs that remain undetected and untreated.^4^ Untreated STIs can lead to multiple adverse sexual and reproductive health outcomes, including increased risk of HIV acquisition, pelvic inflammatory disease, adverse pregnancy and infant outcomes, and infertility.^1^^,^^4^, ^5^, ^6^

In many resource-limited settings (including Kenya), STI control largely relies on syndromic management, where STI treatment is based on the presence of clinical syndromes such as urethral/vaginal discharge and genital ulcer disease rather than laboratory-confirmed diagnosis.^7^ Although syndromic management enables same-day treatment without costly diagnostic tests, it has several limitations.^8^ Most STI cases are asymptomatic, and the inability to test and treat these cases leaves a reservoir of untreated infections and perpetuates persistent untreated infections, leading to continued transmission and long-term adverse health outcomes.^7^^,^^9^ In addition, syndromic management’s reliance on broad-spectrum empiric treatment and the absence of antibiotic susceptibility testing, coupled with the low sensitivity of syndromic algorithms (particularly for vaginal discharge syndromes),^10^^,^^11^ often leads to the overuse of antibiotics, raising serious concerns about antimicrobial resistance, especially for *N. gonorrhoeae*.^8^^,^^11^

Furthermore, reinfection with the same or different STIs is a persistent concern in high STI burden settings. High reinfection rates suggest behavioural, treatment-related, and network factors, such as incomplete partner treatment, poor adherence to multi-day regimens, and ongoing exposure to new or untreated partners^12^^,^^13^ In AGYW, reinfections may also signal a failure to implement effective prevention strategies, including retesting, counselling, or integrated HIV/STI services.^14^ The cycle of recurrent infections may perpetuate adverse health outcomes and sustain a high population-level STI prevalence. Moreover, even when STIs are detected and treated, reproductive tract damage may still occur, with studies suggesting that delayed treatment or repeated infections increase the risk of pelvic inflammatory disease (PID) and other sequelae despite cure of the initial infection.^15^^,^^16^ Although STI reinfection is a recognized issue, most data on the STI burden among AGYW in Kenya and similar settings are derived from cross-sectional surveys, self-reported symptoms, or single-time-point testing. There are limited longitudinal data describing the incidence, persistence, clearance, and recurrence of STIs in this population which are key for targeted STI prevention and control.^4^^,^^17^ Thus, in Kenya, as in many other low income countries, the true burden of STIs, especially among young women, is likely underestimated.^18^

Understanding the STI epidemic among AGYW in Kenya is critical to prioritizing prevention efforts and targeting interventions. To address this knowledge gap, we leveraged longitudinal data from the Kenya Single-dose HPV Vaccine Efficacy Trial (KEN SHE), which enrolled sexually active Kenyan women aged 15–20 years and followed them for a median of 54 months. The study provided laboratory-based STI screening at regular intervals, along with free treatment and risk-reduction counselling. This provides a unique opportunity to closely examine STI incidence, treatment patterns, and recurrence among adolescent girls and young women. This study quantifies the incidence and reinfection rates of bacterial STIs (chlamydia, gonorrhoea, and syphilis), the coverage and concordance of syndromic and etiologic treatment, and the burden of pelvic inflammatory disease in this cohort.

## Methods

### Study design and procedures

The Kenya Single-Dose HPV Vaccine Efficacy (KEN SHE) is an ongoing randomized controlled trial evaluating the efficacy and durability of a single-dose HPV vaccine among young women (clinicaltrials.gov, #NCT03675256). In three clinical trial sites, Thika, Nairobi, and Kisumu, Kenya, serving urban, peri-urban, and surrounding rural catchment populations, the study recruited and enrolled HIV-negative, sexually active young women, with one to five lifetime partners (no minimum recent-activity criterion was applied), aged 15–20, who were not pregnant and had no history of HPV vaccination. This cohort reflects the predominantly Black African composition of the Kenyan population. Ethnicity by sub-national group was not collected as a routine demographic variable. All participants were female, and sex was recorded at enrolment as part of eligibility screening. The study enrolled participants from December 2018 to November 2019, and participants were randomized into three groups: immediate bivalent HPV vaccine and delayed meningococcal vaccine, immediate nonavalent HPV vaccine and delayed meningococcal vaccine, or immediate meningococcal vaccine and delayed nonavalent HPV vaccine. The primary study design and detailed study procedures have been published previously.^19^ The study provided comprehensive sexual and reproductive health services, including contraception, STI testing and treatment, HIV testing and counselling, and pre-exposure prophylaxis (PrEP). HIV testing was conducted at enrolment and at each scheduled six-monthly visit using standard rapid testing algorithms. Participants who seroconverted were referred for care and treatment. At enrolment and study visits, questionnaires were administered to collect data on demographics, sexual and risk behaviour, medical history, and sexually transmitted disease symptoms. Every 6 months, participants received a pelvic and speculum exam with swabs for HPV and STI testing, genital wart inspection, Pap smear, and venous blood collection for syphilis. All questionnaires were administered using electronic case report forms (DF/Net Research, Inc., Seattle, WA, USA). Community engagement was integral to study design and conduct, with a Community Advisory Board at each site providing continuous input on protocol design, recruitment strategies, retention activities, and dissemination plans throughout the study.

### STI testing, treatment and reproductive outcomes assessment

At each quarterly study visit, participants completed a questionnaire on symptoms of sexually transmitted diseases and received counselling on adherence to treatment, risk reduction strategies, and condom use. At enrolment and every 6 months, providers collected blood samples to test for Syphilis, and cervicovaginal swabs to test for bacterial vaginosis (BV), *N. gonorrhoeae* (GC) *and Chlamydia trachomatis* (CT). Syphilis testing used rapid plasma reagin (RPR); reactive samples were confirmed by *Treponema pallidum* haemagglutination assay (TPHA). Quantitative RPR titers were recorded, with titers of 1:8 or higher consistent with active infection per the laboratory algorithm. Nucleic acid amplification tests (APTIMA; Hologic/GenProbe, San Diego, CA) for GC, CT, and Trichomonas vaginalis, and slide scoring (Nugent Score) for BV were performed at the University of Washington, University of Nairobi East Africa, STI Laboratory in Mombasa, Kenya. HSV-2 was tested by type-specific ELISA at enrolment. All laboratory specimens were shipped weekly to the Mombasa laboratory with a 10-day turnaround. All participants reporting symptoms and those with a positive laboratory test for any STI received treatment per Kenya national STI guidelines. Symptomatic participants were treated at the same visit as specimen collection using syndromic algorithms to match symptoms: cervicitis and PID treatments under the cervicitis and lower abdominal pain algorithms; vaginal discharge syndrome treatments based on discharge findings; and vesicular and non-vesicular ulcer treatments under the genital ulcer syndrome algorithm, based on lesion appearance. Participants with positive laboratory test results were treated within four weeks of specimen collection, the typical laboratory turnaround interval. Symptomatic participants or those with positive laboratory test results were encouraged to notify their sexual partners for treatment and received counselling on the importance of treatment adherence, sexual partners’ treatment, and condom use. Partner notification was provider-encouraged and supported by patient-delivered partner therapy: participants with curable bacterial STIs were given a partner treatment pack to deliver to their sexual partner(s). Partner uptake of the offered treatment was assessed by participant self-report at subsequent study visits using a structured partner notification form (yes/no) that recorded whether each named partner accepted and took the prescribed regimen. PID was diagnosed syndromically by trained study clinicians using Kenya national STI guidelines, which require lower abdominal pain plus at least one of cervical motion tenderness, adnexal tenderness, or uterine tenderness on bimanual examination, with or without fever or vaginal/cervical discharge. Clinicians received standardized training on case definition and clinical examination at study initiation and at periodic refresher sessions across the three sites. Pelvic pain was defined as participant-reported STI symptom of lower abdominal pain, abdominal pain, dyspareunia, or any equivalent terms. Chronic pelvic pain was defined as two or more reports of pelvic pain occurring ≥6 months apart. All pregnancies and birth outcomes were documented through routine pregnancy monitoring.

### Statistical analyses

We used descriptive statistics to summarize the demographic and sexual risk characteristics of participants with and without laboratory-confirmed STI diagnoses during KEN SHE study visits occurring on or before June 14, 2024, up to and including visits 18 months post-crossover (throughout this report, baseline refers to the enrolment visit). The primary outcomes were STI incidence, factors associated with STI incidence, reinfection rates, and associated factors. Secondary objectives included concordance between syndromic treatment and laboratory results, as well as reproductive health sequelae.

We first calculated incidence rates for the chlamydia or gonorrhoea (CT/GC) composite, each pathogen individually, and syphilis separately over the follow-up period. Co-infections diagnosed at the same visit, such as simultaneous chlamydia and gonorrhoea, were counted as a single composite CT/GC event. For chlamydia and gonorrhoea, time at risk was measured from enrolment, with each observation interval defined as the time elapsed between consecutive testing visits (from the previous to the current visit). All laboratory-confirmed positive results after enrolment were counted as events, allowing each participant to contribute multiple incident episodes across testing intervals. Time at risk was calculated from baseline to the first post-baseline positive test or to the last visit with a negative laboratory test. Participants who tested positive for chlamydia or gonorrhoea at enrolment were included in the risk set, to prevent prevalent infections from being counted as incident cases; baseline-positive results were excluded from event counts. No formal post-treatment clearance period was applied. The date of the laboratory STI test was assumed to be the date of infection, and tests performed at least 3 months apart were considered distinct infection episodes.

For syphilis, we calculated incidence as time to first infection, excluding participants who tested positive at enrolment, as syphilis serological markers may remain elevated after treatment, making it difficult to distinguish new infection from prior infection. Incident syphilis was defined as the first laboratory-confirmed positive syphilis test (RPR-reactive with TPHA confirmation per the laboratory algorithm) in a participant with a non-reactive result at enrolment. Time at risk was calculated from enrolment to the date of the first positive test, or the last visit with a negative laboratory result if no infection was detected. Incidence rates are expressed per 100 person-years, and 95% confidence intervals for all infections were estimated using Poisson regression. We used Kaplan–Meier methods to plot cumulative incidence for any STI (CT, GC, or syphilis), chlamydia, gonorrhoea, and syphilis over the follow-up period.

We then evaluated baseline characteristics associated with incident CT or GC at any study visit using an Andersen-Gill recurrent events Cox proportional hazards model with robust standard errors and clustering by participant. This approach allowed each participant to contribute multiple STI events while accounting for repeated infections within individuals. Separate models were fitted for the CT/GC composite, gonorrhoea only, and chlamydia only. All models included time-varying covariates updated at each six-month visit: age, bacterial vaginosis diagnosis, self-reported contraceptive use in the past month, number of sexual partners in the past six months, and condom use at last vaginal sex. Contraceptive use was defined as self-reported use of any hormonal or intrauterine contraception (DMPA, OCP, implant, or IUD). These factors were selected a priori based on the STI literature in similar populations.^20^^,^^21^

Lastly, among participants with a confirmed first CT or GC infection, we estimated the incidence of first reinfection for the CT/GC composite and for each pathogen individually. Syphilis was excluded from the reinfection analysis due to the difficulty of distinguishing true reinfection from persistent serological reactivity. Participants with at least one episode of laboratory-confirmed gonorrhoea or chlamydia, including those testing positive at baseline, were included in the reinfection risk set. Time to reinfection was calculated from the date of the first laboratory-confirmed positive result to the date of the first reinfection, defined as a subsequent positive test at least 90 days after the index infection, or to the date of the last study visit if no reinfection occurred. Participants with an index infection of either pathogen were included for the CT/GC composite reinfection. Reinfection was defined as pathogen-specific, requiring a subsequent positive test to match the pathogen of the index infection. Where a participant had index infections of both gonorrhoea and chlamydia, the earliest same-pathogen reinfection event across either pathogen was used. To identify risk factors for reinfection, we used a Cox proportional hazards model with time-varying covariates including age, bacterial vaginosis, contraceptive use, number of sexual partners, and condom use at the last vaginal sex, as well as baseline education level. The model was also adjusted for vaccine arm and study sites.

We used descriptive statistics to summarize the proportion of participants with PID, chronic pelvic pain and ectopic pregnancy. The date of the first PID event on or after month 3 was considered the event date. Participants with no PID event were censored at their last visit. Events occurring within the first three months were excluded from incidence calculations to minimise misclassification of prevalent cases as incident cases. We classified STI exposures into four mutually exclusive groups based on cumulative history of laboratory-confirmed CT or GC and treatment receipt. We defined gold standard treatment as either: (i) adequate syndromic treatment at the same visit as specimen collection (a cervicitis or PID regimen providing CT/GC coverage); or (ii) laboratory-based treatment at a subsequent visit before the next scheduled six-monthly study visit. The four groups were: always gold-standard care (gold-standard treatment at every STI-positive visit), mixed care (those who received gold-standard care at some but not all STI-positive visits), STI without gold-standard treatment (at least one STI but no gold-standard treatment at any positive visit), and no STI, no treatment (reference group, no laboratory-confirmed STI across follow-up). PID incidence rates were calculated as events per 100 person-years, with exact 95% confidence intervals estimated using Poisson regression with a log person-years offset.

All analyses were performed using R, version 4.4.2 (R Core Team (2024), Vienna, Austria https://www.R-project.org/).

### Ethics approval

This study was approved by the Kenya Medical Research Institute (KEMRI) Scientific and Ethics Review Unit (KEMRI/SERU/CMR/P00095/3741 on 26 September 2018 for Kisumu site and KEMRI/SERU/CCR/117/3745 on 19 October 2018 for Nairobi and Thika). All participants provided written informed consent to participate in the study. The study was registered at ClinicalTrials.gov (NCT03675256). Participants aged 15–17 years who were not emancipated minors provided written assent and parent/guardian consent, and emancipated minors and participants aged 18–20 years provided written informed consent per local IRB requirements. The study was conducted in accordance with the principles of the Declaration of Helsinki.

### Role of the funding source

The funders had no role in study design, data collection, data analysis, interpretation of data; writing of the manuscript, or the decision to submit for publication.

## Results

Between December 2018 and November 2019, 2275 young women were enrolled in the KEN SHE study. The median follow-up duration was 54 months (IQR: 52–57 months). Overall, 1355 participants (60%) tested positive at least once for any of chlamydia, gonorrhoea, or syphilis during the study period. Chlamydia was the most common infection, affecting 1233 (54%) participants, followed by gonorrhoea in 440 participants (19%). There were 12 participants (0.5%) with syphilis infection. Participants who were younger (15–17 years) at enrolment (61%), had lower than post-secondary education (61–71%), reported sexual debut before age 18 (60–64%), had two or more sexual partners in the past year (70%), or reported using contraception in the past month (71%) had higher proportions of ever testing positive for any of these three infections (Table 1).

**Table 1:** Baseline sociodemographic and behavioural characteristics by bacterial STI status.

Characteristic	Overall N = 2275 (col %)	Ever tested positive for bacterial STI (CT/GC/syphilis)	
		Never bacterial STI n = 920 (row %)	Ever bacterial STI n = 1355 (row %)
Age (years)^a^			
15–17	1306 (58%)	507 (39%)	799 (61%)
18–20	961 (42%)	411 (43%)	550 (57%)
*Missing*	8	2	6
Education (highest level)			
No schooling	7 (0.3%)	2 (29%)	5 (71%)
Primary school, some or complete	160 (7.0%)	50 (31%)	110 (69%)
Secondary school, some or complete	1654 (73%)	649 (39%)	1005 (61%)
Post-secondary school	454 (20%)	219 (48%)	235 (52%)
Marital status			
Married	96 (4.2%)	38 (40%)	58 (60%)
Never married	2153 (95%)	875 (41%)	1278 (59%)
Other	26 (1.1%)	7 (27%)	19 (73%)
Earns an income of her own	301 (13%)	128 (43%)	173 (57%)
Current smoker	11 (0.5%)	5 (45%)	6 (55%)
Currently consumes alcohol	124 (5.5%)	47 (38%)	77 (62%)
Has a current main or steady sexual partner	1633 (72%)	654 (40%)	979 (60%)
Lives with sexual partner	123 (5.4%)	47 (38%)	76 (62%)
Partner provides financial support	1093 (48%)	424 (39%)	669 (61%)
Age at first vaginal intercourse (years)			
<15	540 (24%)	197 (36%)	343 (64%)
15–17	1281 (56%)	506 (40%)	775 (60%)
≥18	454 (20%)	217 (48%)	237 (52%)
Number of sex partners in last year			
None	469 (21%)	208 (44%)	261 (56%)
One	1604 (71%)	651 (41%)	953 (59%)
Two or more	202 (8.9%)	61 (30%)	141 (70%)
Used condom at last vaginal sex			
No	716 (31%)	279 (39%)	437 (61%)
No vaginal sex	469 (21%)	208 (44%)	261 (56%)
Yes	1090 (48%)	433 (40%)	657 (60%)
Used contraception in the past month^b^	442 (19%)	126 (29%)	316 (71%)

Bacterial STI defined as laboratory-confirmed chlamydia (CT), gonorrhoea (GC), or syphilis at any study visit.

a Missing values: age, n = 8. Percentages calculated using non-missing values as denominator.

b Contraception includes DMPA (depot medroxyprogesterone acetate), IUD (intrauterine device), OCP (oral contraceptive pill), and implants.

### STI incidence

Cumulative incidence of CT, GC and syphilis (composite and individual) is shown in Fig. 1. A total of 2083 new CT or GC events were observed over 9312 person-years, resulting in an overall incidence rate of 22.37 per 100 person-years (95% CI 21.42–23.34). Chlamydia was the most frequent infection, with 1775 new cases over 9312 person-years (incidence rate: 19.06 per 100 person-years; 95% CI 18 .19–19.96), followed by gonorrhoea with 498 new cases over 9312 person-years (5.35 per 100 person-years; 95% CI 4.89–5.83). Ten new cases of syphilis were reported over 9439 person-years (0.11 per 100 person-years; 95% CI 0.05–0.19) (Table 2). Time-to-first-event incidence rates, calculated as time to the first positive test after baseline, are presented in Appendix Table S1.

**Fig. 1:**
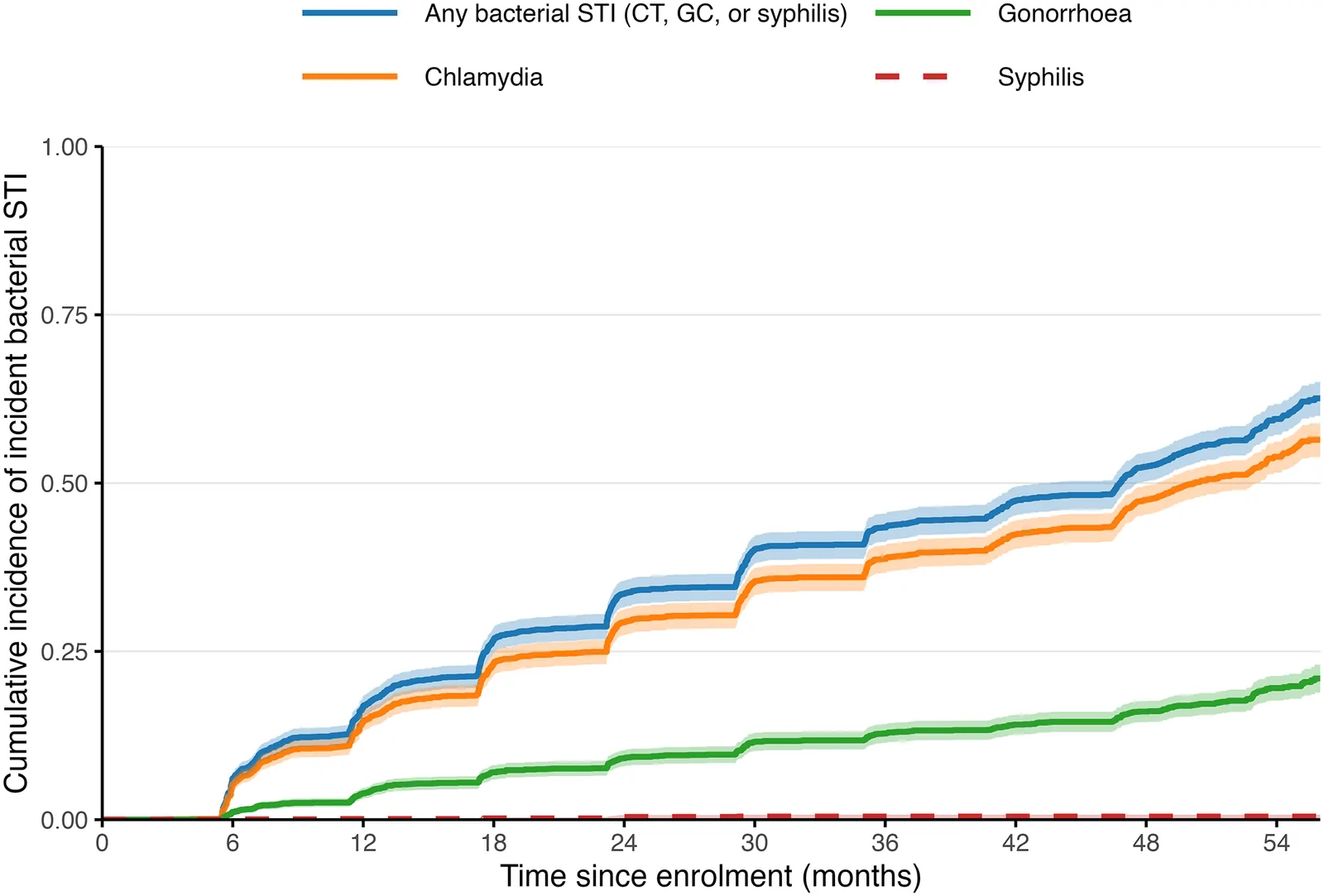
Cumulative incidence of chlamydia, gonorrhoea, and syphilis among Kenyan adolescent girls and young women, KEN SHE cohort. Kaplan–Meier estimates over 54 months of follow-up. Shaded bands represent 95% confidence intervals. STI, sexually transmitted infection.

**Table 2:** Incidence of chlamydia, gonorrhoea, and syphilis among KEN SHE participants.

Bacterial STI	N at risk	Events^a^	Person-years	Incidence rate per 100 person-years^b^ (95% CI)
Any STI (CT or GC)	2275	2083	9312	22.37 (21.42–23.34)
Chlamydia (CT)	2275	1775	9312	19.06 (18.19–19.96)
Gonorrhoea (GC)	2275	498	9312	5.35 (4.89–5.83)
Syphilis	2273	10	9439	0.11 (0.05–0.19)

a All laboratory-confirmed positive results after baseline are counted; each participant may contribute multiple events. CT and GC co-infections are counted as a single bacterial STI event, so CT and GC incidence rates do not sum to the any bacterial STI rate. For syphilis, only the first incident laboratory-confirmed positive result after baseline is counted; participants who tested positive at baseline are excluded from the syphilis analysis.

b Incidence rates and 95% confidence intervals estimated using Poisson regression. Person-years calculated from first study visit across all intervals in the Andersen-Gill counting-process dataset; baseline positive results are excluded from event counts. For syphilis, person-years are calculated from baseline to the first incident positive or last study visit, whichever occurred first.

Results from the Cox proportional hazards model (Table 3) showed that younger age, lower education, BV, contraceptive use, and having one or more sexual partners were associated with a higher risk of incident GC or CT infection. Compared to women with post-secondary education, those with primary education or less had a 34% higher risk of GC or CT diagnosis (aHR: 1.34, 95% CI 1.08–1.67), and those with secondary education had a 23% higher risk (aHR: 1.23, 95% CI 1.03–1.45). Testing positive for BV at the same visit was strongly associated with incident STI (aHR: 1.79, 95% CI 1.63–1.97). Women reporting contraceptive use also had a higher incidence of CT or GC diagnosis (aHR: 1.35, 95% CI 1.22–1.48). Compared to women reporting no sexual partners in the past year, those with one partner had a 45% increased risk (aHR: 1.45, 95% CI 1.25–1.68) and those with two or more partners had an 88% increased risk (aHR: 1.88, 95% CI 1.49–2.37) of CT or GC diagnosis. Similarly, BV, contraceptive use, and multiple sexual partners were each associated with increased risk of both gonorrhoea and chlamydia. Reporting condomless sex at last vaginal intercourse was not significantly associated with incident CT or GC.

**Table 3:** Factors associated with incidence of chlamydia and gonorrhoea among KEN SHE participants.

Characteristic	Any STI (GC or CT)		Gonorrhoea		Chlamydia	
	aHR (95% CI)^a^	p value	aHR (95% CI)^a^	p value	aHR (95% CI)^a^	p value
Age, years^c^	0.84 (0.82, 0.87)	<0.001	0.81 (0.75, 0.87)	<0.001	0.85 (0.82, 0.89)	<0.001
Education, highest level						
Post-secondary school	–	–	–	–	–	–
Primary school or less	1.34 (1.08, 1.67)	0.009	2.38 (1.52, 3.73)	<0.001	1.25 (0.98, 1.58)	0.067
Secondary school, some or complete	1.23 (1.03, 1.45)	0.019	1.87 (1.26, 2.77)	0.002	1.17 (0.98, 1.40)	0.088
Bacterial vaginosis	1.79 (1.63, 1.97)	<0.001	2.07 (1.71, 2.49)	<0.001	1.75 (1.58, 1.94)	<0.001
Contraceptive use in past month^b^	1.35 (1.22, 1.48)	<0.001	1.57 (1.29, 1.93)	<0.001	1.34 (1.21, 1.49)	<0.001
Number of sex partners in last year						
None	–	–	–	–	–	–
One	1.45 (1.25, 1.68)	<0.001	1.54 (1.10, 2.15)	0.012	1.40 (1.19, 1.63)	<0.001
Two or more	1.88 (1.49, 2.37)	<0.001	2.85 (1.81, 4.48)	<0.001	1.73 (1.34, 2.23)	<0.001
Condomless sex at last vaginal sex	0.95 (0.85, 1.06)	0.387	1.08 (0.85, 1.36)	0.537	0.95 (0.84, 1.07)	0.375
Study arm						
*Meningococcal*	–	–	–	–	–	–
Cervarix	1.04 (0.92, 1.17)	0.535	0.99 (0.78, 1.26)	0.943	1.07 (0.94, 1.21)	0.323
GARDASIL 9	1.05 (0.93, 1.19)	0.423	1.07 (0.84, 1.36)	0.581	1.03 (0.90, 1.18)	0.674

aHR = adjusted hazard ratio; CI = confidence interval.

a Adjusted for study site in addition to all variables shown in the table.

b Contraception includes DMPA, IUD, OCP, and implants.

c Time-updated covariates: age, bacterial vaginosis, contraceptive use, number of sex partners, and condomless sex were measured and updated at each scheduled six-monthly visit. Education is a baseline-only covariate.

### Incidence of STI reinfection

Among 1348 participants who had chlamydia or gonorrhoea during the study, 582 (43.2%) had a first reinfection episode with the same pathogen (CT or GC), observed over 2310 person-years of follow-up, resulting in a reinfection incidence rate of 25.20 per 100 person-years (95% CI 23.21–27.30) (Table 4). There were 547 chlamydia reinfection episodes among 1233 participants with a previous chlamydia infection over 2212 person-years of follow-up, resulting in an incidence rate of 25.78 per 100 person-years (95% CI 23.68–28.00). There were also 91 gonorrhoea reinfection episodes among 440 participants with a previous gonorrhoea infection over 825 person-years of follow-up, resulting in a reinfection rate of 11.03 per 100 person-years (95% CI 8.91–13.45). Fig. 2 illustrates the cumulative incidence of first CT or GC reinfection episodes during follow-up. Results from the Cox proportional hazards model showed that younger age (aHR: 0.89, 95% CI 0.83–0.95, p < 0.001), primary school education or less compared to post-secondary education (aHR: 1.44, 95% CI 1.00–2.07, p = 0.050), and contraceptive use (aHR: 1.31, 95% CI 1.10–1.55, p = 0.002) were associated with a higher risk of CT or GC reinfection (Table 5).

**Table 4:** Reinfection incidence of chlamydia and gonorrhoea among KEN SHE participants.

Bacterial STI	Events^a^	Person-years	Reinfection rate per 100 person-years^b^ (95% CI)
Any STI (CT or GC)	582	2310	25.20 (23.21–27.30)
Chlamydia (CT)	547	2122	25.78 (23.68–28.00)
Gonorrhoea (GC)	91	825	11.03 (8.91–13.45)

CT = chlamydia trachomatis; GC = *Neisseria gonorrhoeae*; CI = confidence interval.

a Reinfection is defined as a second laboratory-confirmed positive bacterial STI result at least 90 days after the first (index) infection. Person-time at risk begins at the date of first infection and ends at the date of reinfection or last study visit, whichever occurred first. Only participants with a confirmed first infection are included in this analysis.

b Reinfection rates and 95% confidence intervals estimated using Poisson regression. Syphilis excluded from this table due to insufficient reinfection events as well as difficulty establishing reinfection.

**Fig. 2:**
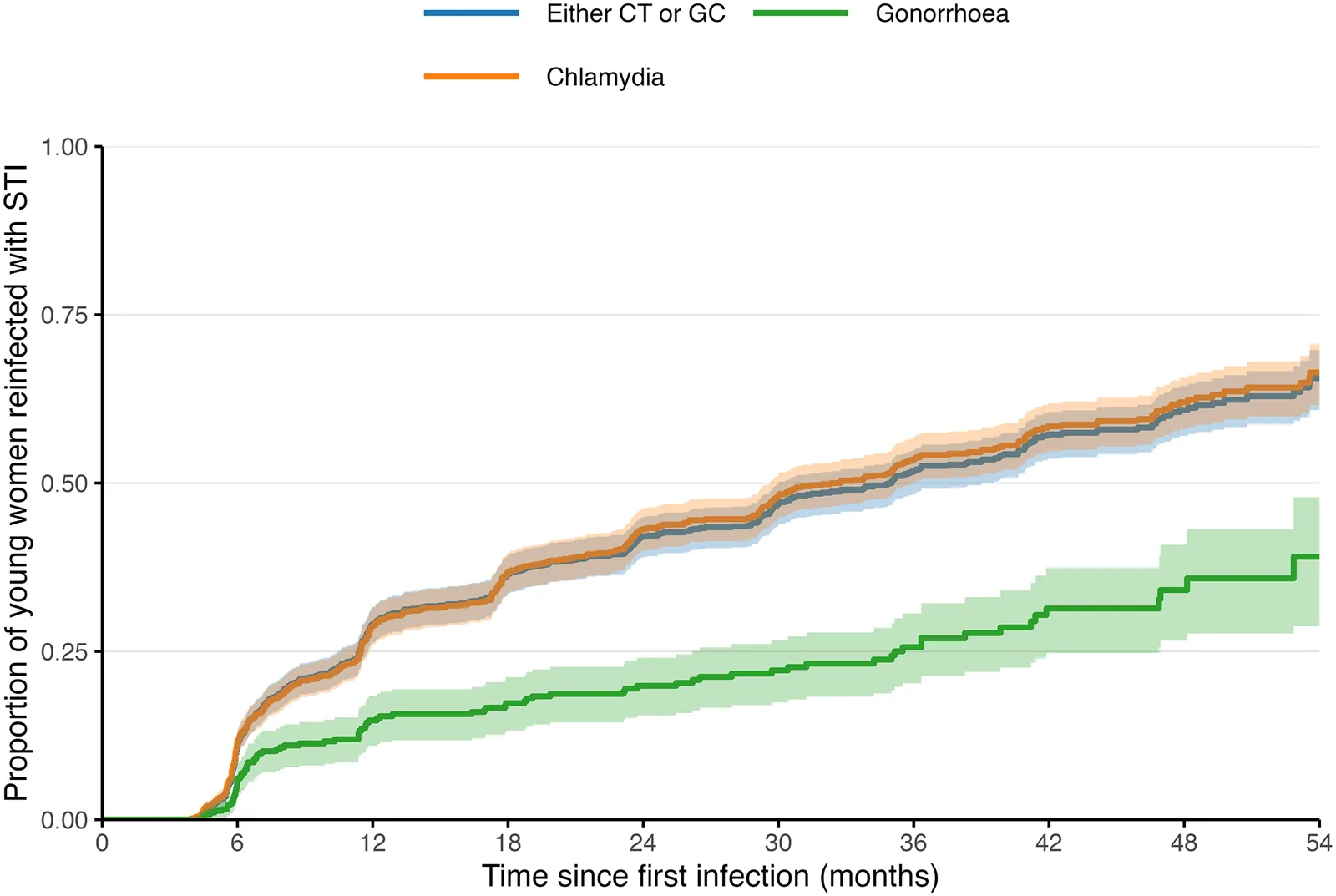
Cumulative incidence of first CT or GC reinfection among participants with a confirmed CT or GC infection, KEN SHE cohort. Curves show the CT or GC composite (blue), chlamydia (orange), and gonorrhoea (green) individually. Kaplan–Meier estimates from the date of the first laboratory-confirmed positive result. Reinfection defined as a subsequent positive test of the same pathogen at least 90 days after the index infection. STI, sexually transmitted infection. Shaded bands represent 95% confidence intervals. STI, sexually transmitted infection.

**Table 5:** Factors associated with reinfection with chlamydia or gonorrhoea.

Characteristic	aHR	95% CI	p-value
Age, years	0.89	0.83–0.95	<0.001
Bacterial vaginosis	1.14	0.96–1.34	0.136
Education, highest level^a^			
Post-secondary school (ref)	–	–	
Primary school or less	1.44	1.00–2.07	0.050
Secondary school, some or complete	1.18	0.89–1.57	0.244
Contraceptive use in past month	1.31	1.10–1.55	0.002
Number of sex partners in last year			
None (ref)	–	–	
One	1.03	0.81–1.33	0.796
Two or more	1.27	0.85–1.89	0.246
Condom use at last vaginal sex			
Protected (ref)	–	–	
No (unprotected sex)	0.87	0.71–1.05	0.152
Study arm			
Meningococcal (ref)	–	–	
Cervarix	1.06	0.87–1.29	0.569
GARDASIL 9	0.99	0.81–1.21	0.922

Cox proportional hazards model with adjustment for site in addition to all variables listed in the table. aHR = adjusted hazard ratio. CI = confidence interval. All covariates are time-varying (updated at each 6-monthly visit) except education.

a Education is a baseline covariate measured at enrolment. Protected = condom used or no vaginal sex reported.

### Coinfection and symptoms

A total of 19,504 visits included laboratory testing for CT, GC, or syphilis, of which 2425 (12.4%) had at least one positive result for CT, GC, or syphilis. Among visits with positive results, 1757 (72.5%) had no reported symptoms. Of the 2425 positive visits, 215 (8.9%) had positive results for multiple bacterial STIs. The most common co-infection was chlamydia and gonorrhoea: among 549 visits with a positive gonorrhoea result, 214 (39.0%) also had chlamydia; among 2071 visits with a positive chlamydia result, 214 (10.3%) also had gonorrhoea. Among the 652 laboratory-positive visits in which symptoms were reported, the most frequently reported symptom was discharge (92%), followed by itching (26%), dysuria (8.7%), and ulcers (2%).

### Treatment

In total, 2114 (92%) participants received at least one episode of STI treatment during the study period. A total of 8623 treatments were administered during the study period, of which 5983 (69%) were syndromic (including PID and other STI syndromes; see Appendix Table S2) and 2640 (31%) were based on laboratory test results. The most frequent syndromic treatment was for vaginal discharge syndrome (78%), followed by cervicitis (12%), PID (6.5%), vesicular ulcers (0.8%), and non-vesicular ulcers (0.4%) (Appendix Table S2). Among lab-based treatments, chlamydia was the most common treatment (54%), followed by bacterial vaginosis (18%), gonorrhoea (15%), trichomonas vaginalis (0.8%), and HSV-2 or syphilis (0.4%). Partner STI treatment or referral was offered at 846 (14%) syndromic-treatment visits and at 1039 (39%) laboratory-based treatment visits. Participants accepted to deliver treatment to their sexual partner(s) at 709 (84%) and 891 (86%) of these visits, respectively.

Among laboratory-confirmed STI-positive tests, 86.5% of chlamydia-positive visits (1774/2050) and 89.7% of gonorrhoea-positive visits (490/546) received adequate treatment before the next scheduled six-monthly testing visit. Approximately 6.3% of chlamydia-positive and 8.4% of gonorrhoea-positive visits received syndromic treatment at the same visit as specimen collection. Of chlamydia-positive visits, 3.3% were treated with a regimen lacking coverage for the pathogen, compared to 2.9% of gonorrhoea-positive visits. Overall, approximately 10% of chlamydia-positive and 7.3% of gonorrhoea-positive visits did not receive treatment within the follow-up window (Appendix Table S3).

### Comparison of syndromic and laboratory-guided treatment approaches

Over the course of the study, there were 3973 six-monthly study visits where participants received treatment based on symptoms and had laboratory testing at the same visit. Of these visits, 2228 (56.5%) had no laboratory-confirmed STI (Fig. 3). Among visits where vaginitis was treated syndromically, 57% had no laboratory-confirmed bacterial STI or BV at the same visit. Similarly, 56% of visits where cervicitis or PID was treated syndromically had no laboratory-confirmed STI at that visit.

**Fig. 3:**
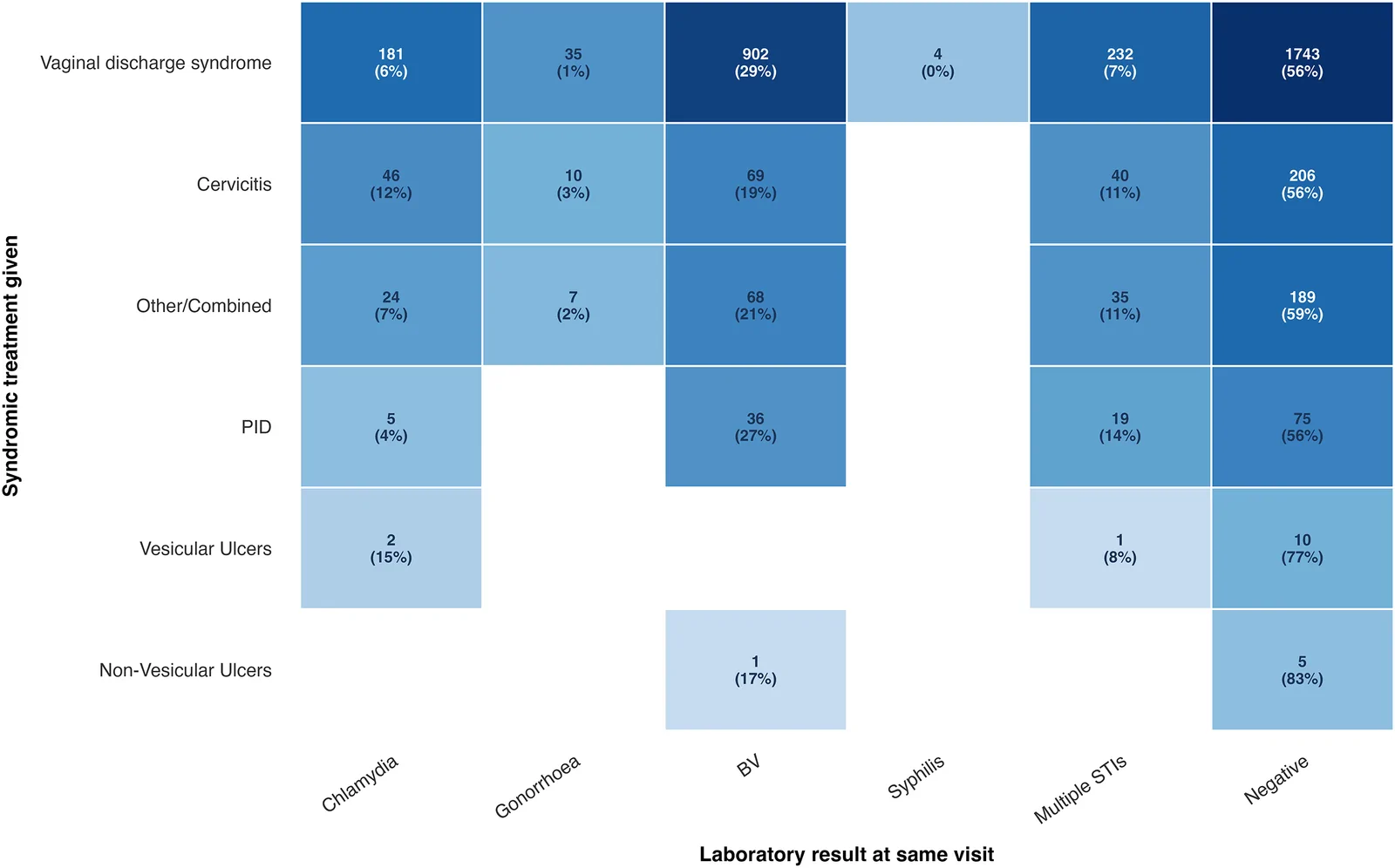
Heatmap of syndromic treatment versus laboratory-confirmed STI status at the same visit. Rows show the syndromic diagnosis treated. Columns show the laboratory result from cervicovaginal swabs and blood collected at the same visit. Cells display visit count and row percentage. Cell shading scales with visit counts. Across vaginal discharge syndrome, cervicitis, other or combined, and PID, 56 to 59 percent of syndromically-treated visits had no laboratory-confirmed STI. Vaginal discharge syndrome row chlamydia (n = 181) and gonorrhoea (n = 35) cells represent undertreatment, as the standard vaginal discharge syndrome regimen lacks coverage for these pathogens. Denominator: 3973 visits. BV, bacterial vaginosis. PID, pelvic inflammatory disease. STI, sexually transmitted infection.

### Reproductive health outcomes

During the study follow-up period, 304 (13.4%) participants received treatment for PID, 12 (0.5%) reported chronic pelvic pain, and 6 (0.3%) experienced an ectopic pregnancy. Among PID cases, 277 incident PID cases were diagnosed at or after three months of follow-up, yielding an overall incidence rate of 3.34 per 100 person-years (95% CI: 2.97–3.75) across 8282.79 person-years of observation. PID incidence varied markedly by laboratory-confirmed CT or GC treatment history (Table 6). Participants who had a laboratory-confirmed STI but never received adequate treatment had the highest incidence at 12.83 per 100 person-years (95% CI: 9.90–16.30). Among those with a history of STI, participants who received gold standard treatment after every CT or GC infection had an incidence of 2.85 per 100 person-years (95% CI: 2.34–3.42), while those with mixed treatment status, receiving adequate treatment at some but not all STI-positive visits, had an incidence of 3.03 per 100 person-years (95% CI: 1.95–4.44). Participants with no laboratory-confirmed STI during follow-up had a comparable incidence of 2.59 per 100 person-years (95% CI: 2.07–3.18).

**Table 6:** PID Incidence by chlamydia or gonorrhoea treatment exposure group.

Group	N	PID Events	Person-Years	Incidence rate (95% CI) per 100 person-years
Overall	2275	277	8282.79	3.34 (2.97–3.75)
Always Gold Standard Care^a^	980	108	3793.16	2.85 (2.34–3.42)
Mixed (Gold Standard + Other)^b^	206	23	759.37	3.03 (1.95–4.44)
CT/GC, No Gold Standard Treatment^c^	162	62	483.16	12.83 (9.90–16.30)
No CT/GC, No Treatment^d^	927	84	3247.10	2.59 (2.07–3.18)

a **Always gold standard care:** Participants with at least one laboratory-confirmed STI (chlamydia or gonorrhoea) who received gold standard treatment for ***every*** CT/GC -positive test before their first PID diagnosis. Gold standard treatment is defined as either: (i) adequate syndromic treatment at the same visit as specimen collection — a cervicitis or PID regimen providing CT/GC coverage; or (ii) laboratory-based treatment at a subsequent visit before the next scheduled 6-monthly study visit or within 6 months of the index lab test.

b **Mixed (gold standard + other):** Participants with at least one laboratory-confirmed CT/GC who received gold standard treatment at ***some but not all*** CT/GC -positive visits before their first PID diagnosis.

c **CT/GC, no gold standard treatment:** Participants with at least one laboratory-confirmed STI who ***never*** received gold standard treatment at any CT/GC -positive visit before their first PID diagnosis. Includes participants who received no treatment or only treatment with inadequate syndromic coverage (e.g., vaginitis or bacterial vaginosis regimen).

d **No STI, no treatment (reference group):** Participants with no laboratory-confirmed CT/GC recorded across all study visits and no gold standard treatment received.

## Discussion

Our findings document a high burden of STIs and associated morbidity in this cohort of Kenyan AGYW. Overall, nearly 60% of participants tested positive for at least one STI (CT, GC, or syphilis), with high rates of reinfection and clinical sequelae, indicating persistent transmission despite regular testing and treatment efforts. These outcomes occurred in the context of regular syndromic treatment, six-monthly laboratory testing and treatment, risk-reduction counselling, and comprehensive sexual health services. The persistent high rates of incidence, reinfection, and reproductive morbidity in a cohort undergoing intensive case management emphasize the importance of comprehensive STI care beyond just testing and treatment in high-burden settings.^13^^,^^22^^,^^23^

Additionally, these persistent high STI rates highlight the urgent need for complementary prevention strategies, including those that operate independently of individual case management. Strengthening partner management remains foundational: comprehensive case management for curable STIs requires effective partner notification and treatment. In our cohort, partner STI treatment or referral was low, and these gaps in partner management likely contribute to the high reinfection rates observed in our cohort. Patient-delivered partner therapy and expedited partner therapy, where regulatory frameworks permit, may help close this gap. Promotion of consistent condom use also remains an evidence-based prevention strategy and should be reinforced alongside biomedical interventions. Evaluation of post-exposure prophylaxis (e.g., doxycycline PEP), pre-exposure prophylaxis, and vaccination strategies is needed, with ongoing Gonorrhoea vaccine trials offering promising solutions.^24^^,^^25^ DoxyPEP has shown promise in MSM populations and in some studies of cisgender women, but recent trials among women, including those in Kisumu, Kenya, have shown mixed results.^24^ Until evidence for biomedical prevention in women strengthens, expanding access to comprehensive case management, partner treatment, and consistent condom promotion remains the most actionable path.

The reproductive sequelae of ongoing STI transmission were evident in our study. We observed a prevalence of PID similar to that reported in other African cohorts, despite having more rigorous STI management than standard care.^4^ Although our study did not evaluate the relationship between recurrent STI episodes and PID, the higher PID incidence among those with untreated infections, coupled with the high rate of STI reinfection, aligns with previous research showing that recurrent chlamydial and gonococcal infections increase the risk of PID through cumulative inflammatory damage.^16^^,^^26^ Although the observed prevalences of chronic pelvic pain and ectopic pregnancy were low, the relatively young age of study participants and limited follow-up time may underestimate the true prevalence of long-term complications as tubal injury and its sequelae, including infertility, could become evident years later.

Our treatment data further illustrate the limitations of current STI management approaches. Antibiotic exposure was nearly universal in this cohort, with the majority of treatment courses delivered under syndromic management. Importantly, the majority of laboratory-confirmed infections were asymptomatic, highlighting the substantial disconnect between symptom-based approaches and disease reality. This mismatch between syndromic treatment approaches and the predominantly asymptomatic nature of STIs represents a structural limitation of symptom-driven case management for asymptomatic infections, which incremental improvements such as refined syndromic algorithms, faster partner notification, or occasional laboratory tests are unlikely to fully resolve. In addition to the high rate of asymptomatic infections, among those treated using syndromic diagnosis and who had laboratory-based tests at the same visit, a substantial proportion tested negative for bacterial STIs and BV, demonstrating both overtreatment and undertreatment inherent in current symptom-based care systems.

Despite our intensive approach of regular syndromic treatment with six-monthly laboratory testing and treatment, reinfection was high, with nearly half experiencing at least one reinfection episode. Although study staff supported same-visit syndromic treatment and offered laboratory-confirmed treatment within four weeks of testing, completion of multi-day regimens (for example, seven-day doxycycline for chlamydia) relied on participant self-administration. We did not measure adherence to treatment. A proportion of episodes classified as reinfections may therefore reflect inadequate clearance of the index infection rather than reacquisition. While augmenting syndromic care with point-of-care diagnostics remains important, our findings suggest testing-and-treatment alone is unlikely to be sufficient for STI control or transmission reduction in high-burden settings. STI control in high-burden settings likely requires comprehensive prevention packages that combine biomedical prevention (vaccines, post-exposure prophylaxis), enhanced diagnostics (point-of-care testing), and structural interventions (partner treatment programmes, community-level screening) deployed simultaneously rather than piecemeal improvements to individual components.

Individual-level risk factors in our study, such as younger age, lower educational attainment, and multiple partners, are consistent with previous research.^27^^,^^28^ However, the high incidence of STIs despite reporting a single steady partner suggests that individual behaviour modification has limited impact in high-prevalence settings and underscores the importance of meso-level factors such as broader community STI prevalence and partner behaviours drive risk independent of individual choices.^29^ It is likely that a high community-level STI burden creates an environment where individuals with lower personal risk behaviours face substantial infection risk due to network effects and partner behaviours beyond their control. The observed association between non-barrier contraceptive use and increased STI risk likely reflects residual confounding by unmeasured aspects of sexual behaviour, partner type, or condomless exposure, rather than a direct causal effect of contraception. Women using non-barrier methods may be more likely to have steady partners with whom condoms are not used, increasing exposure to any STIs. These findings point to the importance of both individual-level and population-level interventions to reduce STI risk in young women.

Our study is one of the largest longitudinal cohorts of AGYW in sub-Saharan Africa, with systematic, laboratory-confirmed STI testing conducted at repeated intervals over more than four years. We provided precise measurements of incident and recurrent infections, overcoming the limitations of syndromic diagnosis or cross-sectional surveys. The study had high retention, detailed behavioural and demographic data, and a pragmatic model integrating STI care into broader sexual health services, which strengthens the applicability of the findings to real-world settings. Finally, the multicenter approach and rigorous analytical methods enhance validity and support the broader relevance of our findings to similar high-burden, low-resource contexts throughout sub-Saharan Africa. However, our study has several limitations. First, our findings reflect a clinical trial cohort receiving comprehensive sexual health services, including quarterly screening and six-monthly laboratory testing. Trial participants may differ from the general Kenyan AGYW population in risk behaviour, healthcare access, and adherence. The persistently high rates, despite this intensive approach, nonetheless suggest that community STI rates, where testing is less frequent and treatment delays are common, may be at least as high. The cohort was also restricted by design to female participants, so disaggregation of findings by sex was not possible; our findings therefore do not speak to the burden, treatment-seeking, or reinfection dynamics of these infections among males. Second, the absence of a comparator arm means we cannot estimate the relative effectiveness of intensified testing-and-treatment versus routine services. Third, laboratory result delays occurred during portions of the study period, potentially extending the time between testing and treatment for some asymptomatic outcomes. Fourth, our reinfection estimates may be conservative or affected by misclassification: six-monthly testing intervals would have missed short-lived or spontaneously resolving infections; the date of a positive test approximated rather than equalled the date of acquisition; and, in the absence of test-of-cure and microbiologic strain typing, some episodes classified as reinfections may represent persistent infection or treatment failure rather than reacquisition. Treatment adherence to multi-day regimens was not directly measured, which adds further uncertainty to the interpretation of reinfection events. Fifth, partner notification and treatment uptake were assessed by participant self-report rather than direct verification of partner attendance, so reinfection rates may reflect, in part, unreported partner-treatment failure or non-uptake despite the availability of patient-delivered partner therapy. Finally, the study was restricted to a limited panel of STIs and did not assess emerging infections (e.g., *Mycoplasma genitalium*) or antimicrobial susceptibility/resistance patterns.

### Conclusions

In a closely monitored cohort of Kenyan AGYW, CT and GC incidence and reinfection remained high, and PID was most common among those with untreated infections, despite intensive testing and treatment. These findings indicate that testing and treatment alone is insufficient for STI control in high-burden settings and that complementary prevention strategies — including strengthened partner management, consistent condom promotion, and biomedical prevention tools with demonstrated efficacy in women — are urgently needed. Future research should shift focus from measuring STI incidence alone to evaluating impact on clinically meaningful health outcomes. The ultimate goal of STI prevention and control should be reducing HIV acquisition, pelvic inflammatory disease, infertility, ectopic pregnancy, and other serious sequelae that disproportionately affect women.^30^, ^31^, ^32^ Studies measuring these downstream health impacts would provide clearer evidence for policy decisions and better guide resource allocation in STI prevention and control programs.

## Contributors

MO and RVB conceptualised the study. MO, ERB, and RVB developed the methodology. MO, KZ, GO, and KBH curated the data. KZ and ERB conducted formal analysis. KZ and ERB created visualisations. MO, NM, IW, EAB, and RVB conducted the investigation. MO, IW, and RVB administered the project. NM, EAB and RVB acquired funding. RVB provided resources. NM, EAB, and RVB supervised the study. MO and KZ wrote the original draft. All authors validated the findings and contributed to review and editing of the manuscript. MO, KZ, ERB and KBH accessed and verified the underlying data. All authors had full access to all data and had final responsibility for the decision to submit for publication.

## Data sharing statement

Research data supporting this publication are available upon reasonable request from the corresponding author and approval from the Kenya Medical Research Institute (KEMRI) Scientific and Ethics Review Unit (SERU), which requires that we release data from Kenyan studies (including de-identified data) only after they have provided their written approval for additional analyses. The application forms and guidelines can be accessed at https://www.kemri.org/seru. To request these data, please get in touch with the corresponding author or the KEMRI SERU at seru@kemri.org.

## Declaration of interests

ERB reports grants from the National Institute of Allergy and Infectious Diseases (NIAID) paid to her institution, and consulting fees from the Infectious Diseases Society of America (IDSA) paid to her for her role as Statistical Editor for the Journal of Infectious Diseases. RVB reports grants from the Bill & Melinda Gates Foundation paid to her institution and an honorarium from Gilead Sciences for service on a Data Monitoring Committee. All other authors declare no competing interests.

## Appendix A Supplementary data

Appendix Tables S1–S3

Supplementary_CONSIDER_KENSHE

EPD form
