# Supplementary material for: Incidence, reinfection, and reproductive morbidity of bacterial sexually transmitted infections among Kenyan adolescent girls and young women receiving systematic testing and treatment: a 4.5-Year prospective cohort analysis
Source: Lancet Reg Health Afr. 2026 Nov;9:None. doi: 10.1016/j.lanafr.2026.100137 (PMC13760030; doi:10.1016/j.lanafr.2026.100137)
Supplement: Supplementary file 1 [file mmc1.pdf]

Supplementary appendix

Incidence, Reinfection, and Reproductive Morbidity of Bacterial Sexually Transmitted Infections Among Kenyan Adolescent Girls and Young Women Receiving Systematic Testing and Treatment: A Prospective Cohort Analysis

TLRHAFRICA-D-25-00255

Table of contents

Appendix Table S1. Time-to-first-event incidence rates of chlamydia, gonorrhoea, or syphilis..................3
Appendix Table S2. Syndromic treatments by visit type among participants receiving syndromic treatment 4
Appendix Table S3. Treatment receipt among laboratory-confirmed STI-positive visits............................5

Appendix Table S1 Time-to-first-event incidence rates of chlamydia, gonorrhoea, or syphilis infections among KEN SHE participants

STI	N at risk	Events[1]	Person-years	Incidence rate per 100 person-years[2] (95% CI)
Any of chlamydia, gonorrhoea, or syphilis	2,275	1,247	6,443	19.35 (18.30–20.45)
Chlamydia (CT)	2,275	1,127	6,776	16.63 (15.68–17.62)
Gonorrhea (GC)	2,275	404	8,502	4.75 (4.30–5.23)
Syphilis	2,273	10	9,439	0.11 (0.05–0.19)

[1] *Incident infection defined as first laboratory-confirmed positive result after baseline. CT and GC co-infection occurred at 8.8% of bacterial STI-positive visits; co-infections counted as a single composite event so CT and GC incidence rates do not sum to the composite rate.*

[2] *Incidence rates and 95% confidence intervals estimated using Poisson regression.*

Appendix Table S2. Syndromic treatments by visit type among participants receiving syndromic treatment (N = 5,983 visits).

	**Overall**	**6-monthly visit (lab screening)**	**Quarterly visit (no lab)**	**Unscheduled/ interim visit**
**Syndromic diagnosis**	N = 5,983	N = 3,973	N = 572	N = 1,438
Vaginal discharge syndrome treatment	4,674 (78%)	3,239 (82%)	435 (76%)	1,000 (70%)
Vaginitis	4,515 (75%)	3,152 (79%)	425 (74%)	938 (65%)
Cervicitis	697 (12%)	503 (13%)	38 (6·6%)	156 (11%)
Pelvic inflammatory disease	386 (6·5%)	148 (3·7%)	35 (6·1%)	203 (14%)
Bacterial vaginosis	149 (2·5%)	81 (2·0%)	9 (1·6%)	59 (4·1%)
Vesicular ulcers	49 (0·8%)	20 (0·5%)	9 (1·6%)	20 (1·4%)
Non-vesicular ulcers	22 (0·4%)	6 (0·2%)	1 (0·2%)	15 (1·0%)
Trichomonas vaginalis	10 (0·2%)	6 (0·2%)	1 (0·2%)	3 (0·2%)
Other	500 (8·4%)	224 (5·6%)	88 (15%)	188 (13%)

Data are n (%). Percentages reflect proportion within each visit-type column. 6-monthly visits included laboratory STI screening. Quarterly visits (other visit codes ending in 0) did not include routine laboratory testing. The vaginal discharge syndrome treatment category combines regimens for bacterial vaginosis, trichomoniasis, and non-specific vaginitis, prescribed empirically because bedside microscopy was not available at study visits.

Appendix Table S3. Treatment receipt among laboratory-confirmed STI-positive visits

STI	Total lab-positive visits (N)*	Adequate syndromic treatment — same visit n (%)	Lab-based treatment — subsequent visit n (%)	Treated — inadequate coverage n (%)	Not treated n (%)
Chlamydia	2,051	129 (6.3%)	1,645 (80.2%)	68 (3.3%)	209 (10.2%)
Gonorrhea	546	46 (8.4%)	444 (81.3%)	16 (2.9%)	40 (7.3%)
Syphilis	19	0 (0%)	15 (78.9%)	1 (5.3%)	3 (15.8%)

Adequate syndromic treatment: cervicitis (cefixime/ceftriaxone/gentamicin + azithromycin) or PID (cefixime/ceftriaxone/gentamicin + doxycycline) regimen given at the same visit as specimen collection, where the participant was symptomatic. These regimens provide coverage against chlamydia and gonorrhoea. Non-vesicular ulcer treatment (Benzathine Penicillin + azithromycin) is considered adequate for syphilis.

Lab-based treatment: any treatment (syndromic or lab-based) recorded at any visit between the positive laboratory visit, up to and including either the next scheduled 6-monthly visit or 6 calendar months after the positive test, whichever was later

Treated — inadequate coverage: any treatment given at the same visit as specimen collection that would not provide coverage against the detected pathogen (e.g., vaginitis treatment with clotrimazole/metronidazole for a chlamydia-positive visit).

Not treated: no treatment recorded at the positive laboratory visit or at any subsequent visit up to the next 6-monthly scheduled visit.

***Excluded lab positive visits** where participant reached the data cutoff visit (visit code 8180) before the treatment window closed; treatment status could not be determined.
