# Supplementary material for: Incidence, reinfection, and reproductive morbidity of bacterial sexually transmitted infections among Kenyan adolescent girls and young women receiving systematic testing and treatment: a 4.5-Year prospective cohort analysis
Source: Lancet Reg Health Afr. 2026 Nov;9:None. doi: 10.1016/j.lanafr.2026.100137 (PMC13760030; doi:10.1016/j.lanafr.2026.100137)
Supplement: Supplementary file 2 [file mmc3.pdf]

Equitable Partnership Declaration

If any questions do not apply to your study, please indicate "N/A" for "not applicable.
For more information on how to complete this form see the Information for Authors document.

Researcher considerations

1. Please detail the involvement that researchers who are based in the country or countries of study had during a) study design; b) clinical study processes, such as processing blood samples, prescribing medication, or patient recruitment; c) data interpretation; and d) manuscript preparation, commenting on all aspects. If they were not involved in any of these aspects, please explain why.

   This should include a thorough description of their leadership roles in the study. Are local researchers named in the author list or the acknowledgements, or are they not mentioned at all (and, if not, why)? Please also describe the involvement of early career researchers based in the location of the study. Some of this information might be repeated from the Contributors section in the manuscript. Note: we adhere to ICMJE authorship criteria for naming authors on a paper.

**a) Study design:**<br><br>MO (Kenya) and RVB co-conceptualised this nested analysis. The parent KEN SHE trial was designed with leadership from Kenyan investigators (MO, NM, EAB) at KEMRI. Local investigators determined STI outcomes as priority given high burden in Kenyan AGYW.
**b) Clinical study processes:**<br><br>All clinical procedures were conducted by Kenyan staff at three Kenyan sites (Thika, Nairobi, Kisumu). MO, GO and IW (Kenya-based early career researchers) led participant recruitment, sample collection, and clinical assessments. Laboratory testing was performed at University of Nairobi/University of Washington STI Laboratory in Mombasa, Kenya.
**c) Data interpretation:**<br>MO (Kenya) led interpretation with KZ, GO, and KBH (early career researchers) and EAB and NM (Kenya senior career researchers). Findings were contextualised through Kenyan investigators' understanding of local health systems, syndromic management practices, and AGYW-specific barriers.
**d) Manuscript preparation:**<br>MO (Kenya, early career) drafted the manuscript with KZ (early career). All Kenyan co-authors (MO, NM, GO, IW, EAB—five of nine authors) reviewed, edited, and approved the final version. MO is lead and corresponding author.

2. How was funding used to remunerate and enhance the skills of researchers in the countries of study? And how was funding used to improve research infrastructure at the study sites?

   Potentially effective investments into long-term skills and opportunities within local institutions could include training or mentorship in analytical techniques and manuscript writing,

opportunities to lead all or specific aspects of the study, financial remuneration rather than requiring volunteers, and other professional development and educational opportunities.

Improvements to research infrastructure could include funding extended trial designs (eg, platform trials), establishment of long-term contracts for research staff, building research facilities, and setting up local control of funding allocation.

**Skills:**<br>Early career Kenyan researchers (MO, GO, IW) received training in epidemiological methods, data management, and scientific writing. KZ received mentorship in biostatistical analysis. Study provided opportunities for MO to lead manuscript development and serve as corresponding author.
**Research infrastructure:**<br><br>Funding supported clinical research capacity at three Kenyan sites in Nairobi, Thika and Kisumu and lab capacity in the Mombasa STI lab, including equipment for specimen collection, testing, storage and electronic data capture systems. Staff were provided with long-term staff contracts throughout the 54-month follow-up.

3. How did you safeguard the researchers who implemented the study?

   Please describe how you guaranteed safe working conditions for study staff, including provision of appropriate personal protective equipment, protection from violence, and prevention of overworking.

Study staff received standard occupational health protections including PPE for specimen handling. We provided regular supervision and manageable caseloads to prevent overworking and burnout. Research staff were salaried employees with benefits and not volunteers.

Benefits to the communities and regions of study

4. How does the study address the research and policy priorities of its location?

   How were the local priorities determined and then used to inform the research question? Who decided which priorities to take forward? Which elements of the study address those priorities?

STI control is a Kenya Ministry of Health priority. Kenya's National STI Strategic Plan identifies AGYW as a key population requiring targeted interventions. Local investigators (MO, NM, EAB) identified the research question based on clinical observations of high STI burden despite syndromic management thus addressing a gap in longitudinal Kenyan and broader African data.

5. How will research products be shared in the community of study?

   For instance, will you be providing written or oral layperson summaries for non-academic information sharing? Will study data be made available to institutions in the region(s) of study? The Lancet Global Health *encourages authors to translate the summary (abstract) into relevant languages after paper editing; do you intend to translate your summary?*

   Findings will be shared with Kenya Ministry of Health and county health departments of Kisumu, Thika and Nairobi. Lay summaries will be prepared for participants at study sites and community health promoters within catchment areas of the study sites. We intend to translate the summary into Kiswahili and Dholuo after publication. Study data will remain accessible to KEMRI investigators.

6. How were individuals, communities, and environments protected from harm?

a) *How did you ensure that sensitive patient data were handled safely and respectfully? Was there any potential for stigma or discrimination against participants arising from any of the procedures or outcomes of the study?*

   STI results were confidential and disclosed only to participants. Data were stored on password-protected servers with identifying information separated from clinical data. We addressed STI-related stigma through private counselling and participant education. We encouraged partner notification, but its uptake was voluntary.

b) *Might any of the tests be experienced as invasive or culturally insensitive?*

   Pelvic examinations and cervicovaginal swabs were performed by trained female clinicians in private settings. The procedures were explained beforehand. Participants could decline any examination or opt for self-sampling. All procedures aligned with standard clinical care in the Kenyan health setting.

c) *How did you determine that work was sensitive to traditions, restrictions, and considerations of all cultural and religious groups in the study population?*

   Study procedures were developed with Kenyan investigators familiar with local norms. Throughout we had Community advisory board which provided input and informed recruitment approaches and procedures. The CAB had diverse membership across genders, ages, geography, socio-economic status, religions and culture. Because Kisumu is culturally different from Thika and Nairobi, we had 2 separate CABs domiciled in both regions respectively. Furthermore, our clinic hours accommodated school schedules for adolescent participants.

d) *Were biowaste and radioactive waste disposed of in accordance with local laws?*

Yes. Biological specimens were disposed per Kenya Environmental Management and Coordination Act requirements and institutional biosafety protocols.

e) *Were any structures built that would have impacted members of the community or the environment (such as handwashing facilities in a public space)? If so, how did you ensure that you had appropriate community buy-in?*

N/A. No structures were built. The study was conducted within existing KEMRI research facilities.

f) *How might the study have impacted existing health-care resources (such as staff workloads, use of equipment that is typically employed elsewhere, or reallocation of public funds)?*

N/A. The study was conducted in dedicated research facilities, not public health clinics. There was no diversion of clinical staff, equipment, or public funds from routine healthcare services.

7. Confirm that local ethics review was sought, and please provide the approval number. If not sought, please explain why.

The study was approved by Kenya Medical Research Institute (KEMRI) Scientific and Ethics Review Unit (SERU) as KEMRI/SERU/CMR/P00095/3741 for Kisumu site and KEMRI/SERU/CCR/117/3745 for Nairobi and Thika.

Secondary analyses

8. Have the data analysed in your study been extracted from another source, such as a national survey, rather than being directly collected by the authors of this paper?

No

If the authors of this paper were not involved in data collection, how were the findings interpreted with sufficient contextual knowledge?
The Lancet Global Health *believe contextual understanding is crucial for informed data analysis and interpretation.*

N/A. Data was collected directly by the authors as part of the KEN SHE trial. All Kenyan investigators were involved from study inception through analysis.

---

9. Please provide the title (eg, Dr/Prof, Mr/Mrs/Ms/Mx), name, and email address of an author who can be contacted about this statement.

Name: Dr. Maricianah Onono
Email: maricianah@gmail.com; monono@kemri.go.ke

10. Finally, please provide the title and name of an author from one country of study who has seen and approved this form.

Name: Dr. Maricianah Onono
