# Supplementary material for: Incidence, reinfection, and reproductive morbidity of bacterial sexually transmitted infections among Kenyan adolescent girls and young women receiving systematic testing and treatment: a 4.5-Year prospective cohort analysis
Source: Lancet Reg Health Afr. 2026 Nov;9:None. doi: 10.1016/j.lanafr.2026.100137 (PMC13760030; doi:10.1016/j.lanafr.2026.100137)
Supplement: Supplementary file 3 [file mmc2.docx]

**Supplementary Material: CONSIDER Statement Completion**

| **Item** | **CONSIDER checklist item** | **KEN SHE substudy response** |
| --- | --- | --- |
| **Governance** | | |
| 1 | Partnership agreements between research institution and Indigenous-governing organisation (e.g., MOU, MOA) | KEN SHE was implemented by the Kenya Medical Research Institute (KEMRI). The CONSIDER concept of agreements with an Indigenous-governing organisation does not directly apply to this study population. |
| 2 | Accountability and review mechanisms for harm minimisation | Study conduct was reviewed by the KEMRI Scientific and Ethics Review Unit (SERU, Ref 3741) with periodic safety and progress reporting. A Community Advisory Board (CAB) at each study site contributed to ongoing community accountability. |
| 3 | Protection of Indigenous intellectual property and knowledge | The CONSIDER concept of Indigenous IP and traditional knowledge protection is not directly applicable to this study population. |
| **Prioritisation** | | |
| 4 | How research aims emerged from priorities of Indigenous stakeholders, governing bodies, funders, and empirical evidence | The substudy emerged from the high observed STI prevalence and incidence in the parent KEN SHE HPV vaccine trial, identified gaps in longitudinal STI data among adolescent girls and young women (AGYW) in sub-Saharan Africa, and consultation with Kenyan investigators and county health authorities on priority research questions for adolescent reproductive health. |
| **Relationships (stakeholders and research team)** | | |
| 5 | Adherence to Indigenous ethical guidelines, processes, and approvals | All study protocols were approved by KEMRI SERU. Administrative approval was also obtained from the respective County Departments of Health and Education. All methods were performed in accordance with the Declaration of Helsinki and relevant Kenyan and international research guidelines. |
| 6 | Involvement of stakeholders in research processes (design, funding, implementation, analysis, dissemination, recruitment) | A Community Advisory Board at each of the three study sites provided continuous input on recruitment strategies, retention activities, package of comprehensive reproductive health services to be provided and dissemination plans throughout the study. County health authorities provided input on integration with local services and sustainability |
| 7 | Expertise of research team in Indigenous health and research | The substudy was led by Kenyan investigators based at KEMRI with established expertise in AGYW sexual and reproductive health research in Kenyan settings. The multidisciplinary team included epidemiologists, clinicians, social scientists, and laboratory scientists with experience conducting AGYW research in Kenya. |
| **Methodologies** | | |
| 8 | Methodological approach including rationale and implications for privacy and confidentiality | This was an observational cohort analysis nested within a randomised HPV vaccine trial, using Andersen-Gill recurrent events Cox proportional hazards models with robust standard errors. Individual confidentiality protections were maintained throughout data collection, analysis, and reporting. Analysis was done on de-identified data. Detailed methods are described in the main manuscript. |
| 9 | Consideration of physical, social, economic, and cultural environment | Analysis and interpretation considered the role of gender power asymmetries in partner negotiations, healthcare access constraints, economic constraints on healthcare utilisation, and stigma in shaping STI risk and treatment-seeking behaviour. |
| **Participation** | | |
| 10 | Individual and collective consent for future analyses on samples and data | Participants provided written informed consent or assent at enrolment for primary study activities and for storage of biological samples for future research use. Any further secondary analyses or third-party sample access require additional ethics approval. |
| 11 | Resource demands on participants and communities | Participants received reimbursement for travel and time at each study visit, and received comprehensive sexual and reproductive health services (contraception, STI testing and treatment, HIV testing and counselling, and HIV pre-exposure prophylaxis) as a benefit of participation. CAB members received reimbursement for their time and expertise. |
| 12 | Storage, removal, and disposal of biological samples and data | Biological samples were processed and stored at KEMRI laboratories in Kenya under standard biobanking procedures. Storage and disposal followed Kenyan national guidelines for biological materials. No samples were removed from Kenya without specific ethics approval. Data are stored at KEMRI under restricted access, with sharing governed by the data availability statement in the main manuscript. |
| **Capacity** | | |
| 13 | Support for development of Indigenous research capacity | The study built research capacity in Kenya through training and mentorship of Kenyan junior investigators, clinical research coordinators, data managers, and laboratory staff. Kenyan investigators led the substudy and the resulting publications. |
| 14 | Professional development of research team for partnership | Team members participated in cross-institutional training in research methods, ethics, and community engagement. Mentorship across the international research team supported bidirectional skills transfer. |
| **Analysis and interpretation** | | |
| 15 | Critical inquiry and strength-based approach inclusive of stakeholder values | Findings are interpreted within the structural context of AGYW sexual and reproductive health in Kenya. The high participant retention is evidence of the cohort's engagement with research rather than as evidence of individual or population-level deficit. |
| **Dissemination** | | |
| 16 | Dissemination of findings to relevant governing bodies and peoples | Findings are shared with CAB members, county health authorities, and study participants through community forums at each site, alongside presentations at Kenyan and international scientific conferences and publication in peer-reviewed journals. |
| 17 | Knowledge translation and implementation to support advancement | Findings will inform ongoing engagement with Kenya's Ministry of Health on STI management approaches for AGYW and contribute to national and global discussion on biomedical STI prevention strategies. Engagement with national STI guideline review processes is ongoing. |
